# Fotemustine-based therapy in combination with rituximab as a first-line induction chemotherapy followed by WBRT for newly diagnosed primary central nervous system lymphoma: a prospective phase II trial

**DOI:** 10.20892/j.issn.2095-3941.2021.0026

**Published:** 2021-10-12

**Authors:** Jingjing Wu, Fenghua Gao, Wenhua Wang, Xudong Zhang, Meng Dong, Lei Zhang, Xin Li, Ling Li, Zhenchang Sun, Xinhua Wang, Xiaorui Fu, Linan Zhu, Mengjie Ding, Songtao Niu, Zhaoming Li, Yu Chang, Feifei Nan, Jiaqian Yan, Hui Yu, Xiaolong Wu, Zhiyuan Zhou, Jieming Zhang, Mingzhi Zhang

**Affiliations:** 1 Cancer Research Institute, Department of Oncology, The First Affiliated Hospital of Zhengzhou University, Zhengzhou 450052, Henan, China

**Keywords:** Rituximab, primary central nervous system lymphoma, pemetrexed, fotemustine, WBRT

## Abstract

**Objective:**

This study aimed to evaluate the safety, efficacy, and feasibility of the rituximab, fotemustine, pemetrexed, and dexamethasone (R-FPD) regimen followed by whole-brain radiotherapy (WBRT) for patients with primary central nervous system lymphoma (PCNSL).

**Methods:**

A prospective, single-center phase II clinical trial was conducted. Patients with PCNSL newly diagnosed at the First Affiliated Hospital of Zhengzhou University between July 2018 and July 2020 were studied. The R-FPD regimen consisted of rituximab (375 mg/m^2^ i.v. on D0), fotemustine (100 mg/m^2^ i.v. on D1), pemetrexed (600 mg/m^2^ i.v. on D1), and dexamethasone (40 mg i.v. on D1-5). Patients 60 years or younger who showed a complete response (CR) were treated with 23.4 Gy of WBRT after the end of chemotherapy; those older than 60 years with CR were treated with a wait-and-see approach; and those who did not show CR after the 4th cycle of chemotherapy were given salvage WBRT 30 Gy + local tumor field irradiation up to 45 Gy, regardless of age.

**Results:**

A total of 30 patients were included. After 2 cycles, the objective response rate (ORR) was 96.5% (28/29, 1 CR, 27 PR, 0 SD, and 1 PD). After 4 cycles, the ORR was 73.1% (19/26, 11 CR, 8 PR, 4 SD, and 3 PD). After WBRT, the ORR was 90.9% (10/11, 7 CR, 3 PR, and 1 SD). The grade III and IV toxicity responses were mainly leukopenia (20.0%), thrombocytopenia (23.3%), and anemia (10.0%).

**Conclusions:**

Fotemustine-based therapy in combination with rituximab chemotherapy followed by WBRT can improve outcomes, providing ORR benefits and favorable tolerability in patients newly diagnosed with PCNSL.

## Introduction

Primary central nervous system lymphoma (PCNSL) is a rare but highly aggressive subtype of non-Hodgkin lymphoma affecting exclusively the central nervous system, including the brain parenchyma, spinal cord, leptomeninges, cerebrospinal fluid (CSF), and eyes. More than 90% of PCNSL cases are diffuse large B-cell lymphoma (DLBCL) with a nongerminal center B-cell origin, thus potentially partly explaining its poor prognosis^1^. Rarely, PCNSL can be a T cell, Burkitt, lymphoblastic, or marginal zone lymphoma^2^. Immunodeficiency, like human immunodeficiency virus infection, organ transplantation, and immunosuppressive agents, is a clear risk factor for PCNSL. In recent years, the incidence of immunocompetency in patients with PCNSL has increased nearly 3-fold^3^. According to consensus opinion, the treatment of PCNSL should include 2 stages: induction chemotherapy and consolidation therapy^4^. The induction phase is mainly comprehensive chemotherapy. Most regimens include drugs able to cross the blood-brain barrier (BBB) at conventional doses, such as steroids and some alkylating agents. Cytostatics, such as methotrexate and cytarabine, which have a low-to-moderate ability to cross the BBB, can be safely administered at high doses to increase CNS bioavailability^5^. High-dose methotrexate (HD-MTX) in combination with high-dose cytarabine followed by whole-brain radiotherapy (WBRT) is now considered the standard treatment^6^. However, the HD-MTX treatment-associated mortality rate is 2%–7%, the incidence of grade 3 or 4 nephrotoxicity or stomatitis is 10%; 7%–10% of patients discontinue treatment, and 26%–44% of patients experience a decline in renal function. The dose of aminopterin is decreased in response. In addition, HD-MTX requires active folic acid detoxification, urine alkalization, and hydration management in the hospital, thus resulting in limited use of the drug in older patients. Therefore, identifying alternative treatments for PCNSL is an urgent clinical need^7^.

Fotemustine, a cytotoxic alkylating agent belonging to the nitrosourea family, is highly fat soluble and easily crosses the BBB. The concentration in the cerebrospinal fluid can reach 30%–50% of the plasma concentration. On the basis of its pharmacokinetic properties, fotemustine has been used in the treatment of primary or metastatic brain tumors^8^. Pemetrexed is an antifolate preparation that inhibits the enzyme necessary for the synthesis of folic acid. The drug is similar in structure to methotrexate and can also penetrate the central nervous system. Pemetrexed inhibits cell replication by disrupting folate-dependent metabolic processes in the cell, thus inhibiting tumor growth^9^.

In our previous prospective randomized study, we evaluated the tolerability and efficacy of fotemustine-based polychemotherapy compared with high-dose methotrexate plus cytarabine therapy in patients with newly diagnosed primary CNS lymphoma. Of the 49 eligible patients, no significant difference was observed in terms of the objective response rate (ORR), 2-year progression-free survival (PFS) rate, or 3-year overall survival (OS) rate. The group receiving high-dose methotrexate plus cytarabine therapy showed more serious neutropenia (*P* = 0.009) than the group receiving fotemustine-based treatment^10^. The addition of rituximab to conventional chemotherapy significantly improved the prognosis of systemic DLBCL, the most common form of PCNSL. Accordingly, we combined the fotemustine, pemetrexed, and dexamethasone regimen with rituximab to create an immunochemotherapy protocol for patients with PCNSL. Herein, we report the results of a prospective single-center pilot trial investigating the feasibility and efficacy of this new immunochemotherapy in patients with newly diagnosed PCNSL.

## Materials and methods

### Eligibility criteria

This prospective study enrolled patients newly diagnosed with PCNSL meeting the following criteria: age 14–80 years; KPS score ≥ 40 or Eastern Cooperative Oncology Group (ECOG) score ≤ 3; expected survival time longer than 3 months; CD20 positivity; immunocompetency; newly diagnosed PCNSL according to the World Health Organization (WHO) criteria; treatment at the Lymphoma Center of the First Affiliated Hospital of Zhengzhou University; absence of contraindications to chemotherapy (hemogram and physiological examination results < 7 days); at least one measurable lesion according to RECIST standards; no other serious diseases conflicting with the treatment plan; feasibility of follow-up; no use of other anti-tumor drugs at different times during the treatment period, such as bisphosphonate anti-bone metastasis treatment and other symptomatic treatments; and patient understanding of the goal the study and provision of signed informed consent. Histopathology was conducted by pathologists at the above provincial hospitals. The study was registered with the Ethics Committee of the First Affiliated Hospital of Zhengzhou University (Approval No. SS-2019-035) and at ClinicalTrials.gov (No. NCT04083066). All patients or guardians signed an informed consent form before participating in the study.

### Study design and treatment protocol

This trial was performed as an open, prospective, single-center clinical study. From July 2018 to July 2020, 30 patients were enrolled consecutively. No participants or researchers involved in the study were blinded to treatment assignment. Medication was administered according to the following protocol: rituximab 375 mg/m^2^, micropump pumped for 4 h on day 0; fotemustine 100 mg/m^2^, 1 h infusion on day 1; pemetrexed 600 mg/m^2^, > 0.5 h infusion on day 1; and dexamethasone 40 mg, 1 h infusion on days 1–5. Intravenous mannitol (125 mL, 25 mg) was administered before rituximab. PEG-rhG-CSF (100 µg/kg) was administered subcutaneously starting 48 h after the end of therapy. Blood work was performed every 3 days, and patients were given a subcutaneous injection of granulocyte CSF (5 mcg/kg/d) until the ANC reached ≥ 500/L for 2 days or ≥ 1,500/L for 1 day. Fluoroquinolone antibiotics for bacterial prophylaxis were initiated at ANC < 500/L and continued until the ANC reached ≥ 500/L. If fever occurred during antibiotic treatment or if the chest CT showed fungal infection, fungal infection prophylaxis was given until the body temperature decreased to normal for 3 consecutive days and the findings of the CT were negative. Preventive measures against herpes simplex virus and varicella-zoster virus included ganciclovir or acyclovir. Management of hyperthermia and neutropenia, and blood transfusion support were performed according to institutional guidelines. During chemotherapy, the intrathecal injection of chemotherapy drugs was performed as follows: CSF protein negative: intrathecal injection of MTX 12 mg + Ara-C 50 mg + Dex 5 mg on day 2; CSF protein elevated: intrathecal injection of MTX 12 mg + Ara-C 50 mg + Dex 5 mg on day 2 and day 7. Cycles were repeated every 21 days, and a maximum of 4 cycles was planned unless evidence of progressive disease (PD) or significant toxicity contraindicating treatment continuation were found. After the end of the 2nd and 4th cycles, the patients’ clinical responses were evaluated on the basis of enhanced MRI scans, and adverse reactions were recorded. After 4 cycles of chemotherapy, patients 60 years or younger with a complete response (CR) were treated with 23.4 Gy of WBRT after the end of chemotherapy; those older than 60 years and showing CR were treated with a wait-and-see approach; and those who did not show CR after the 4th cycle of chemotherapy were given salvage WBRT 30 Gy + local tumor field irradiation up to 45 Gy, regardless of age. This study complied with the Declaration of Helsinki.

### Evaluation of response

Baseline MRI was obtained for all patients before therapy initiation. The assessment of response was based on changes in the tumor size of enhanced lesions on MRI, and was performed on the 2nd and 4th cycles and after WBRT, according to the modified International PCNSL Collaborative Group (IPCG) Response Criteria^11^. CR was defined by the complete disappearance of the lesion; PR was defined by a 50% or greater reduction in tumor size; SD was defined by a lesion volume reduction of < 50%, a lesion enlargement of < 25%, or a state between partial remission and disease progression; and PD was defined as an increase in lesion volume by more than 25% or the appearance of a new lesion. The treatment efficacy was evaluated at the end of the 2nd and 4th cycles, and adverse reactions were recorded. Mini-mental status examination (MMSE) and evaluation of adverse reactions were performed at baseline before each treatment cycle and with each follow-up evaluation. Treatment toxicity was graded according to the National Cancer Institute Common Terminology Criteria for Adverse Events (CTCAE, version 3.0)^12^. Follow-up visits occurred in the first month after the end of chemotherapy, every 3 months in the first year, and every 6 months for the next 4 years. A review was performed once per year thereafter.

### Prognostic analysis

Prognostic factors including Bcl-2, MYC proto-oncogene (c-Myc), ECOG performance score, age, CSF protein concentration, serum lactate dehydrogenase (LDH) level, and deep brain involvement have been demonstrated to be meaningful in previous studies. Two pathologists who were blinded to the clinical data interpreted the immunohistochemical labeling independently. All patient samples were scored by 2 observers without knowledge of patient outcomes.

### Statistical analysis

The primary study endpoints were the ORR, PFS, and OS. The secondary endpoint was adverse drug reactions. OS was defined as the time from initial diagnosis until death from any cause, and PFS was defined as the time from diagnosis to disease recurrence, disease progression, or death. PFS and OS were estimated with the Kaplan-Meier method with 95% CIs. Statistical analyses were performed in SPSS version 22 (SPSS, Inc., Chicago, IL, USA).

## Results

Thirty patients were enrolled from July 2018 to July 2020, including 11 men and 19 women, with a median age of 56 years (range 18–76 years). The basic characteristics of the patients are summarized in **Table 1**. Eighty percent of patients had MMSE scores of 27 or above at enrollment, values considered cognitively normal. All patients had evaluable disease. In terms of immunohistochemistry, all patients had CD20-positive DLBCL.

**Table 1:** Clinical characteristics of 30 patients

Patient ID	Gender/age	Pathological type	KPS	Ki-67	LDH	CSF protein	C-MYC	Bcl-2 Bcl-6
1	M/67	DLBCL	80	80%	Normal	Elevated	50%+	90% + Positive
2	F/71	DLBCL	80	80%	Normal	Elevated	40+	90% + Positive
3	F/56	DLBCL	40	85%	Normal	Elevated	50%+	90% + Positive
4	F/54	DLBCL	70	80%	Elevated	Negative	50%+	80% + Positive
5	F/50	DLBCL	80	90%	Normal	Negative	20%+	20% + Positive
6	M/46	DLBCL	40	70%	Normal	Elevated	50%+	80% + 80%+
7	M/64	DLBCL	90	50%	Normal	Negative	40%+	80% + Positive
8	F/64	DLBCL	80	90%	Normal	Elevated	40%+	40% + Positive
9	M/63	DLBCL	90	70%	Normal	Elevated	60%+	80% + Positive
10	F/54	DLBCL	90	90%	Elevated	Negative	30%+	30% + Positive
11	F/56	DLBCL	90	70%	Normal	Negative	40%+	70% + Positive
12	M/64	DLBCL	70	70%	Elevated	Elevated	30%+	40% + Positive
13	F/70	DLBCL	70	90%	Normal	Negative	40%+	30% + Positive
14	F/54	DLBCL	80	80%	Elevated	Elevated	30%+	5% + Positive
15	M/55	DLBCL	90	90%	Normal	Elevated	50%+	20% + Positive
16	M/55	DLBCL	70	80%	Elevated	Elevated	20%+	10% + 60%+
17	F/60	DLBCL	80	80%	Elevated	Elevated	60%+	90% + 90%+
18	M/76	DLBCL	80	90%	Elevated	Negative	Negative	20% + Negative
19	M/75	DLBCL	80	70%	Normal	Negative	20%+	60% + Positive
20	F/56	DLBCL	90	40%	Elevated	Negative	Negative	40% + Negative
21	F/70	DLBCL	70	80%	Elevated	Negative	30%+	90% + 40%+
22	F/69	DLBCL	80	90%	Elevated	Negative	30%+	80% + 70%+
23	F/56	DLBCL	90	80%	Elevated	Elevated	Negative	90% + 70%+
24	F/58	DLBCL	80	90%	Elevated	Elevated	80%+	80% + Positive
25	M/18	DLBCL	90	80%	Elevated	Negative	30%+	20% + 60%+
26	F/58	DLBCL	90	70%	Elevated	Elevated	60%+	90% + 80%+
27	F/43	DLBCL	90	90%	Normal	Elevated	80%+	20% + Positive
28	M/46	DLBCL	90	80%	Elevated	Elevated	80%+	70% + Positive
29	F/51	DLBCL	80	60%	Elevated	Elevated	30%+	30% + Positive
30	F/46	DLBCL	80	90%	Elevated	Negative	30%+	70% + 70%+

### Response and outcomes

After 2 cycles of treatment, 1 patient died of PD before the second cycle of treatment; 1 patient died of pneumonia; and 1 patient died of pneumonia, arrhythmia, and electrolyte disturbance. A total of 29 patients completed the second cycle. After 4 cycles of treatment, in addition to the above 3 patients, another patient (patient 24) died of disease progression before the 4th-cycle evaluation. A total of 26 patients completed the 4th cycle, and 11 patients underwent WBRT.

**Figure 1:**
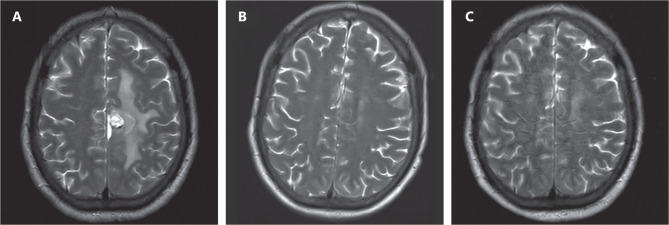
(A) MRI image of a patient with lymphoma in the right frontal and parietal area before treatment. (B) MRI showing marked tumor shrinkage after 2 cycles of treatment. (C) MRI showing tumor disappearance after 4 cycles of treatment. (According to our evaluation criteria, the size of the lesion was approximately 1.5 × 2.0 cm before chemotherapy; the size of the lesion was 0.9 × 0.8 cm after 2 cycles; the efficacy was evaluated as partial remission; the lesion disappeared after 4 cycles; and the efficacy was evaluated as complete remission.)

**Figure 2:**
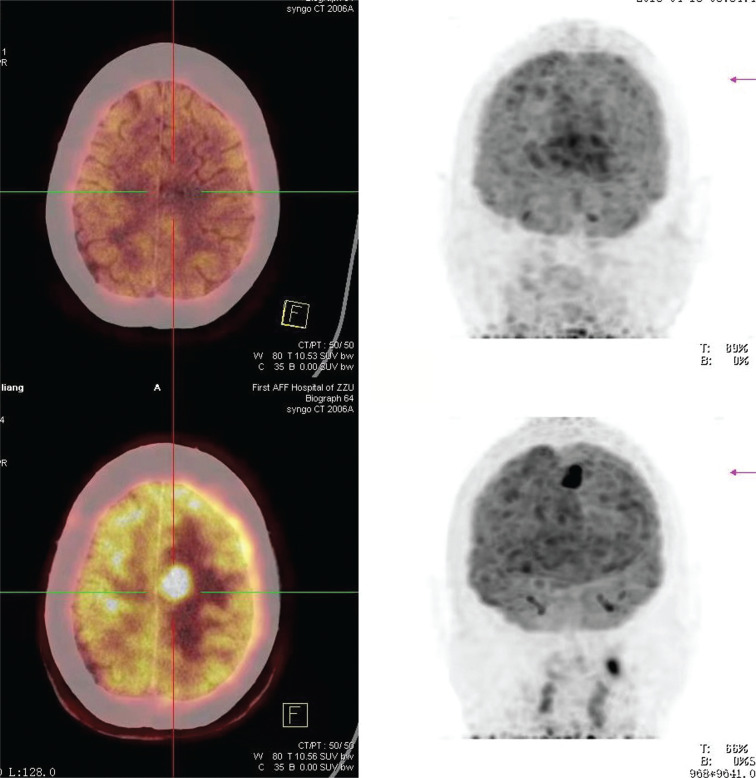
Before treatment, there was a high-density nodule in the near midline of the left frontal lobe, and the lesion disappeared after treatment.

At the end of the 2nd cycle, 27 of 29 (93.1%) had PR, whereas 1 of 29 (3.4%) had CR, with an ORR (CR% and PR%) of 96.5%. An overall response was observed in 19 of 26 patients (73.1%), 11 (42.3%) of whom had CR, and 8 (30.8%) of whom achieved PR at the end of the 4th cycle. **Figure 1** shows the imaging changes in patient 11 before treatment and after the 2nd cycle and 4th cycle, and **Figure 2** shows the comparison of PET-CT images before and after treatment.

**Figure 3:**
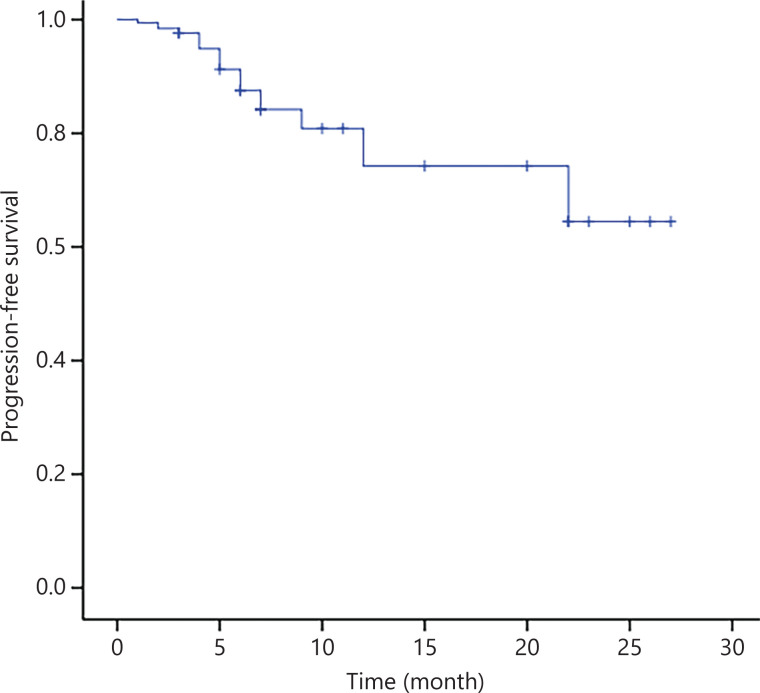
PFS of 30 patients with PCNSL treated with the R-FPD regimen. Patients with no observed outcome are marked as bars in the graphs.

**Figure 4:**
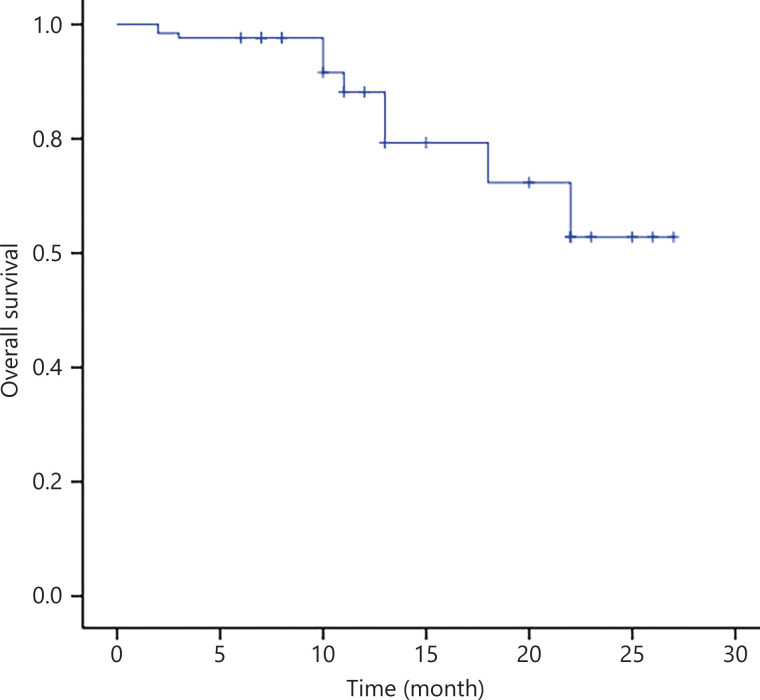
OS of 30 patients with PCNSL treated with the R-FPD regimen. Patients with no observed outcome are marked as bars in the graphs.

After WBRT, 3 of 11 patients (27.3%) had PR, whereas 7 of 11 patients (63.6%) had CR, with an ORR of 90.9% (**Table 2**). For eligible patients, i.e., those with a median follow-up of 11 months, the median PFS and OS were not reached (**Figures 3 and 4**).

**Table 2:** Responses to the R-FPD regimen

Response	Patients *n*	CR *n*(%)	PR *n*(%)	SD *n*(%)	PD *n*(%)	ORR (%)
After C2	29	1(3.4)	27(93.1)	0	1(3.4)	96.5
After C4	26	11(42.3)	8(30.8)	4(15.4)	3(11.5)	73.1
After WBRT	11	7(63.6)	3(27.3)	1(9.0)	0	90.9

### Toxicity

The main toxicity responses are detailed in **Table 3**. Toxicity was mainly associated with myelosuppression, with 20.0%, 23.2%, and 10.0% of patients exhibiting grade III or IV leukopenia, thrombocytopenia, and anemia, respectively. Most nonhematological toxicity symptoms were mild and transient, including digestive tract toxicity, hepatic dysfunction, peripheral nervous system symptoms, and electrolyte imbalance. After symptomatic treatment, the symptoms were alleviated and disappeared. Two deaths (6.7%) associated with infection and 2 deaths (6.7%) associated with disease progression were observed during R-FPD treatment. During follow-up, 5 of 30 patients (16.7%) died of several different causes; the cause of death was PCNSL as a relapse from CR (*n* = 3) and PR (*n* = 1), and 1 patient with CR died of pneumonia. There were no statistically significant changes in MMSE over time. However, these data may be limited by the high number of patients who progressed (9/30) or were censored (2/30) and were no longer evaluated. After the completion of WBRT, only 7 patients completed an MMSE.

**Table 3:** Main adverse reactions in 30 patients treated with the R-FPD regimen

Toxicity	No toxicity observed, *n*(%)	Grade of adverse reaction *n*(%)			
		I	II	III	IV
Hematologic					
Leukopenia	16(53.3)	1(3.3)	7(23.3)	5(16.7)	1(3.3)
Thrombocytopenia	18(60.0)	2(6.7)	3(10.0)	3(10.0)	4(13.3)
Anemia	13(43.3)	9(30.0)	5(16.7)	2(6.7)	1(3.3)
Nonhematologic					
Infection	20(66.7)	2(6.7)	5(16.7)	1(3.3)	2(6.7)
Digestive tract toxicity	18(60.0)	3(10.0)	7(23.3)	2(6.7)	0
Hepatic dysfunction	19(63.3)	9(30.0)	2(6.7)	0	0
Peripheral nervous system symptoms	24(80.0)	6(20.0)	0	0	0
Electrolyte imbalance	19(63.3)	6(20.0)	3(10.0)	1(3.3)	1(3.3)

## Discussion

The diagnosis and treatment of PCNSL is difficult, and the prognosis is poor. In recent years, the incidence rate has shown a clear increasing trend, thus posing major challenges. To date, this is the first prospective, single-center trial investigating the feasibility and efficacy of the new immunochemotherapy R-FPD in patients with newly diagnosed PCNSL. The preliminary results of this study indicate that the R-FPD regimen might be a feasible and effective treatment for newly diagnosed PCNSL.

An analysis of SEER research data^3^ showed that the median survival time of untreated patients with PCNSL was only 1.5 to 3.3 months, and the 5-year OS rate of patients receiving comprehensive treatment was 25% to 42%. This finding suggests that PCNSL has a poor prognosis and short survival. For conventional chemotherapy, HD-MTX-based combination chemotherapy, which can penetrate the BBB, is usually chosen^13,14^. The IELSG32^15^ trial included 227 patients with newly diagnosed PCNSL, of whom 69 were in the methotrexate–cytarabine plus rituximab (R-MA) group. At the end of chemoimmunotherapy, 21 (30%) of the 69 patients achieved CR, with an ORR of 74% in the group. However, the incidences of grade III or IV neutropenia and thrombocytopenia were 56% and 74%, respectively. Three patients died of treatment-related toxicity. Roth et al.^16^, in a clinical trial assessing the outcomes of older patients with PCNSL, indicated an ORR for HD-MTX-based chemotherapy of 44% in older patients (age 70 or above) and 57% in younger patients. Toxicity was age independent, except for a higher rate of grade III or IV leukopenia observed in the older patients (34% *vs.* 21%). Death during treatment was more frequent in older than younger patients (18% *vs.* 11%), and the PFS and OS were shorter in older than younger patients (4.0 *vs.* 7.7 months and 12.5 *vs*. 26.2 months, respectively). Promisingly, this study prospectively enrolled 30 patients with PCNSL. At the end of R-FPD chemoimmunotherapy, the CR rate was 42.3%, and the ORR was 73.1%. In terms of adverse effects, the grade III or IV toxicity responses were mainly leukopenia (20.0%), thrombocytopenia (23.3%), and anemia (10.0%). Only one patient (3%) died of treatment-related toxicity. Compared with previous studies, this program achieved a better CR rate, with a lower incidence of hematological toxicity and treatment-related mortality. At a median follow-up of 11 months, relapse after CR had been achieved in 4 of 12 (33.3%) patients. Four patients (40%) achieved only PR (*n* = 10) and developed PD. As of the follow-up date, 14 patients had sustained remission. Most of the patients (*n* = 5) who experienced relapse received at least 2 therapy courses according to the protocol, and the duration of CR ranged from 1 to 19 months. Interestingly, among the patients achieving CR after WBRT (*n* = 11), only 2 experienced relapse. Therefore, we carefully conclude that the combination of chemotherapy and radiotherapy might have prognostic value. Unfortunately, the number of patients analyzed was small, and extensive statistical data are not available to evaluate the effect of the combination of chemotherapy and radiotherapy on the duration of CR. For eligible patients, although the median PFS and OS were not reached, the survival curves of PFS and OS indicated that patient prognosis can be predicted.

The application of rituximab and fotemustine was associated with significant improvements in the complete remission rate and ORR without higher rates of hematological toxicity or severe complications. The addition of rituximab to conventional chemotherapy has significantly improved the prognosis of systemic DLBCL, the most common form of PCNSL^17–19^. Beyond the commonly used R-MA regimen, this study added fotemustine to pemetrexed instead of MTX. Fotemustine (Muphoran) is an anticancer drug that can penetrate the CNS, and has experimentally been shown to have substantial antitumor activity. The drug has been approved for the treatment of malignant metastatic melanoma and primary brain tumors^20^. A clinical trial has reported that 8 (33%) of 24 patients achieved CR, and 13 patients achieved PR, with an ORR of 88% in fotemustine-based chemotherapy^10^. Although this study used multiple drugs, the results may indicate that fotemustine is an effective and well tolerated drug for PCNSL treatment. Studies have shown that, compared with MTX, pemetrexed has more targets; is simpler to apply; and does not require hydration, urine alkalization, or detoxification. These characteristics make pemetrexed an ideal alternative to MTX. A retrospective study has suggested that pemetrexed may be useful as salvage therapy in recurrent PCNSL; an ORR of 64.7% was found, and all patients achieved CR with a PFS of 5.8 months^21^. Several studies have shown that PCNSL occurs primarily in older patients with a median age of approximately 67 years^22^. The median age of the patients included in our study was 56 years, which was younger than the age in previous studies and may have been associated with the small number of enrolled cases. The incidence has increased in the past 20–30 years, particularly among older patients, thus drawing widespread attention. However, among older patients, HD-MTX-based therapy has low disease control rates and high treatment-related mortality^16^. For older patients who cannot tolerate MTX toxicity, the R-FPD regimen is a better choice. We recommend R-FPD as an alternative to R-MA in patients with high risk of adverse effects. Further studies, such as randomized controlled trials and studies with larger sample sizes, are needed to verify our research results.

Consolidation therapy includes WBRT and high-dose chemotherapy plus autologous stem cell transplantation (HCT-ASCT). A prospective phase II study^23^ has evaluated the efficacy and toxicity of WBRT and HCT-ASCT as first-line consolidation therapy. A total of 140 patients were enrolled. The 2-year PFS rates of the WBRT and ASCT groups were 63% (95% CI, 49%–81%) and 87% (95% CI, 77%–98%), respectively. The WBRT group had a tendency to show increased neurotoxicity, including psychomotor slowdown, neurocognitive impairment, memory dysfunction, gait ataxia, and behavioral changes; in addition, the recurrence rate was high. Cognitive function was retained or improved after ASCT, in line with findings from other studies^24,25^. In our study, WBRT was well tolerated, and the adverse effects were mostly mild (grade 1/2) peripheral nervous system symptoms. At the end of the follow-up, no treatment-related late effects were observed. As defined in our study protocol, patients 60 years or younger who showed CR were treated with 23.4 Gy of WBRT after the end of chemotherapy; those older than 60 years who showed CR were treated with a wait-and-see approach. The way that WBRT is applied in this regimen may be clinically relevant to the lower incidence of neurotoxicity observed. Undoubtedly, as time passes, delayed radiotherapy-related adverse effects, particularly neurocognitive decline, gradually appear and must be carefully documented and addressed. The IELSG32^5^ trial has proposed that ASCT should be the first choice for young patients with PCNSL. Our center will discuss the efficacy and toxicity of WBRT or HCT-ASCT as a first-line consolidation treatment for PCNSL in the future.

Some aspects of this trial merit discussion. First, the open-label design might have introduced an assessment bias, with potentially unbalanced assessment of efficacy or tolerability among patients. Second, this trial is a single-center, single-arm study lacking control groups. The clinical trial was originally designed as a randomized controlled study, but because of patient concerns about the toxicity of methotrexate, enrolling patients in the R-MAD group was difficult, and only 1 patient had been enrolled by the time of follow-up. The choice of control groups for an international randomized trial in primary CNS lymphoma is a challenging issue because of the lack of consensus regarding the standard first-line regimen. Various HD methotrexate-based regimens have been assessed in trials, including combinations with rituximab/temozolomide^26,27^, rituximab/cytarabine (R-MA)^6,28^, rituximab/thiotepa/cytarabine (MATRix)^29^, or rituximab/vincristine/procarbazine^13,30^.

The IELSG scoring system is widely used in the prognostic assessment of patients with PCNSL. The IELSG score includes the ECOG performance score, age, CSF protein concentration, serum LDH level, and deep brain involvement to determine prognosis^31^. Another widely used scoring system is the Memorial Sloan-Kettering Cancer Center score^32^, based on 2 parameters (KPS and age). However, a consensus on prognostic scoring systems in PCNSL remains lacking. With the development of biomolecular technology, more researchers have begun to explore prognostic biomolecular markers for patients with PCNSL. Several studies have indicated that the double-hit of MYC and Bcl-2 gene involvement and the dual expression of MYC and Bcl-2 proteins in systematic DLBCL are associated with poor prognosis^33,34^. However, the significance of MYC and Bcl-2 protein expression in PCNSL is not yet fully clear. Our study showed that age, CSF protein, ECOG score, and high BCL-2 protein expression were not significantly associated with clinical outcomes. LHD level, deep brain invasion, and high MYC protein expression were associated with a significantly increased risk of death and lower OS (**Table 4**). A study has shown that MYC expression, detected by immunohistochemistry, is associated with poor prognosis and may increase the accuracy of risk stratification of patients with PCNSL^35^, in agreement with our research results. Nevertheless, another study on 59 patients with PCNSL has demonstrated that neither MYC expression (with or without BCL-2 coexpression) nor other variables are predictive of clinical outcomes^36^. Given the small number of patients enrolled in this study and the short follow-up time, the number of cases must be increased, and the follow-up time must be extended to further and more accurately explore the clinical significance of MYC protein expression in PCNSL.

**Table 4:** Prognostic factors

Factor	No. (%)	mPFS	*P value	mOS	*P value
Total patients	30 (100)	NA	NA	NA	NA
Older than or equal to 60 y	10 (33.3)	Not reached	0.582	Not reached	0.577
Elevated LDH	15 (50.0)	12.0	0.0001	18.0	0.0001
Elevated CSF protein	11 (36.7)	Not reached	0.085	Not reached	0.088
Deep brain parenchyma involvement	19 (63.3)	Not reached	0.0001	Not reached	0.0001
ECOG ≥ 2	16 (53.3)	Not reached	0.255	Not reached	0.124
MYC > 40%	13 (43.3)	Not reached	0.021	Not reached	0.100
BCL-2 > 50%	19 (63.3)	Not reached	0.067	Not reached	0.262

*P < 0.05 was considered statistically significant.

In conclusion, this prospective phase II study met the primary endpoint and demonstrated that fotemustine-based therapy in combination with rituximab chemotherapy may be an alternative protocol for patients with PCNSL. The regimen may improve outcomes, providing PFS and OS benefits, while improving tolerability for patients newly diagnosed with PCNSL. However, the ORR was not significantly improved with respect to those in previous studies, possibly because of the small number of patients enrolled and the short follow-up time. Therefore, more patients must be included, the follow-up time should be extended, and further randomized controlled trials are needed to confirm our observations.
